# In Vitro Cyto- and Genotoxicity Assessment of Antibacterial Paints with Triclosan and Isoborneol

**DOI:** 10.3390/toxics10020058

**Published:** 2022-01-27

**Authors:** Micaela Machado Querido, Fernanda Rosário, Maria João Bessa, Francisca Mendes, José Carlos Teixeira, João Paulo Teixeira, Cristiana Costa Pereira

**Affiliations:** 1 Environmental Health Department, National Institute of Health, 4000-055 Porto, Portugal; micaelaquerido@hotmail.com (M.M.Q.); fe.8rosario@gmail.com (F.R.); mjbessa8@gmail.com (M.J.B.); franciscairmendes@gmail.com (F.M.); josecarlos_1997@hotmail.com (J.C.T.); cristiana.pereira@insa.min-saude.pt (C.C.P.); 2 EPIUnit, Institute of Public Health, University of Porto, 4050-600 Porto, Portugal; 3 Laboratory for Integrative and Translational Research in Population Health (ITR), 4050-600 Porto, Portugal; 4 Instituto de Ciências Biomédicas Abel Salazar, University of Porto, 4050-313 Porto, Portugal

**Keywords:** antibacterial, paint, triclosan, isoborneol, cytotoxicity, genotoxicity

## Abstract

Surfaces with antimicrobial properties are gaining notoriety as an efficient method to avoid surface contamination. Self-disinfecting paints are a promising strategy towards cleaner indoor environments by preventing the colonization of walls with microorganisms. However, its widespread use needs an appropriate toxicological safety evaluation due to the potential for biological disturbance associated to its biocidal activity. In this work, the cyto- and genotoxic assessment of two self-disinfecting paints containing the antimicrobial substances triclosan (TCS) and isoborneol (ISB) is performed. HaCaT and A549 cell lines models were selected for the in vitro assessment. To evaluate the cytotoxicity, tests by direct contact and on extracts obtained from leaching were performed following ISO 10993, whereas the genotoxicity was assessed by comet assay and cytokinesis-block micronucleus (CBMN) assay. The results showed low levels of cyto- and genotoxicity under the models and conditions tested, indicating that these substances have commercial potential.

## 1. Introduction

Nosocomial infections are a huge problem, causing tremendous economic and human costs every year across the world. One of the major causes contributing to this problem is infection spreading through surface contamination [1]. The persistent contamination of surfaces on healthcare settings is closely related to the high prevalence of microorganisms on these facilities, and innumerous research studies have confirmed the prevalence of pathogens on hospital surfaces, frequently even after regular cleaning procedures are applied [2].

Hospital walls and ceilings are among the more often contaminated surfaces [3] and represent a potential route of infection spreading, since these surfaces are less regularly cleaned comparing to other hospital surfaces, such as bed rails or medical devices [4].

During the last years, several strategies have been proposed to develop surfaces with antimicrobial or anti-adhesive properties, namely paints with antimicrobial properties [5]. The B-SAFECOAT research project consortia recently developed and tested a technology to immobilize antimicrobial substances on a commercial water-based paint. This strategy allowed us to obtain self-disinfecting paints with antibacterial properties aiming to be applied in healthcare settings. The self-disinfecting paints, containing either triclosan (TCS) at 0.0012 g/L or isoborneol (ISB) at 1.2 g/L, two substances with known antimicrobial properties, proved their high antibacterial activity against Gram-positive and Gram-negative bacteria [6]. The published results demonstrate that both paints present potential to be applied as an effective strategy to prevent surface contamination.

Although there are some published studies involving paints with antimicrobial efficacy, so far there are only few studies presenting a robust evaluation of paints’ toxicity. Furthermore, the majority of studies involving self-disinfecting paints concern antifouling paints applied as protective coatings on ship hulls to prevent aquatic organisms’ adhesion and colonization. This being true, most of these studies evaluate paints’ toxicity towards aquatic organisms, such as algae [7,8], with none directed to higher trophic levels.

The developed self-disinfecting paints aimed at indoor environments present a risk of exposure to workers applying the paint and to individuals coming into contact with it after application, including patients and medical staff. Therefore, this work aims at evaluating potential toxic effects from exposure to the developed paints.

The in vitro cyto- and genotoxicity of the self-disinfecting paints, containing TCS or ISB, were assessed in two cell models: human keratinocytes (HaCaT) and human alveolar epithelial (A549). These cellular models represent two of main routes of exposure to paints, inhalation and absorption through direct contact with skin. To carry out this toxicologic analysis in a more realistic approach, simulating the exposures with the final features of the paints when applied in surfaces, tests involving direct contact were performed as well as on extracts obtained from leaching samples painted with the developed paints in order to analyze any dose-related toxicities. Moreover, the conducted cytotoxicity assays—water-soluble tetrazolium (WST-1) cell proliferation assay, neutral red uptake (NRU) and lactate dehydrogenase (LDH) cytotoxicity assay—were based on ISO 10993, which regulates biological evaluation of medical devices [9] to ensure obtaining results coherent to its anticipated use.

## 2. Materials and Methods

### 2.1. Chemicals

Dulbecco’s modified Eagle’s medium (DMEM) with 4.5 g/L glucose and 2 mM L-glutamine and Trypsin-ethylenediaminetetraacetic acid (Trypsin-EDTA) 0.25%/1 mM EDTA2Na in Hank’s balanced salt solution (HBSS), w/o:Ca and Mg, w:Phenol red were acquired from PanBiotech (Aidenbach, Germany). Antibiotic-antimycotic (100×) solution and phosphate-buffered saline (PBS) 10× Molecular Biology Grade were purchased from Corning (Corning, NY, USA). Fetal bovine serum heat inactivated (FBS) was bought from Biowest (Nuaille, France). Triton X-100 (CAS No. 9002-93-1), low melting point (LMP) agarose (CAS No. 39346-81-1), ethylenediaminetetraacetic acid disodium salt dihydrate (Na_2_EDTA) (CAS No. 6381-92-6), Neutral Red (CAS No. 553-24-2) and acridine orange (CAS No. 494-38-2) were purchased from Sigma-Aldrich (St. Louis, Jefferson, MO, USA). The water-soluble tetrazolium (WST-1) cell proliferation reagent kit (CAS No.150849-52-8) and lactate dehydrogenase (LDH) cytotoxicity detection kit were purchased from Roche (Basel, Switzerland). Sodium chloride (NaCl) (CAS No. 7647-14-5) and (Invitrogen, Waltham, MA, USA) TM SYBR^®^ Gold solution were bought from Thermo Fisher Scientific (Waltham, Massachusetts (MA), USA). Normal melting point (NMP) agarose was supplied by Bioline (London, UK). Dimethyl sulfoxide (DMSO) (CAS No. 67-68-5) was purchased from Honeywell (Seelze, Germany). Tris hydrochloride (Tris HCl) (CAS No.1185-53-1), tris base (CAS No. 77-86-1), sodium hydroxide (NaOH) (CAS No. 1310-73-2), methyl methanesulfonate (MMS) (CAS No. 66-27-3) and sodium lauryl sulfate (SLS) (CAS No. 151-21-3) were bought from Merck KGaA (Darmstadt, Germany). Cytochalasin B was bought from PanReac (Barcelona, Spain).

### 2.2. Cell Culture

A549 cell line, a human alveolar pulmunary cell line (ECACC 86012804; Human Caucasian lung carcinoma) was acquired from the European Collection of Authenticated Cell Cultures (ECACC, Salisbury, UK).

HaCaT cell line, a nontumorigenic immortalized human keratinocyte cell line was obtained from Cell Lines Service (Eppelheim, Germany).

Both cell lines were cultured in complete medium (DMEM supplemented with 10% (*v*/*v*) FBS and 1% (*v*/*v*) antibiotic-antimycotic solution) at 37 °C, 5% CO_2_ in a humidified atmosphere. For cell culture maintenance, the medium was changed three times a week and the cultures were split, using 0.25% trypsin-EDTA, when 80% confluency was reached.

### 2.3. Samples Preparation

The paints were prepared and characterized in the previous work by Querido et al. [6]. The samples used in the tests by direct contact and on extracts were the unmodified commercial water-based paint (Un_Paint) and the self-disinfecting paints containing TCS in the concentration 0.0012 g/L and ISB in the concentration of 1.2 g/L applied in a 10 × 10 mm polymeric film square. For the tests by direct contact, samples of transparent polymeric film (W) were used as the negative control of the surface and copper (Cu^2+^) was used as the positive control.

Before each test, every sample was sterilized with UV-C light (294 nm) using a UV lamp from VWR (Radnor, PA, USA) for 15 min on each side.

### 2.4. Tests by Direct Contact

**Figure 1:**
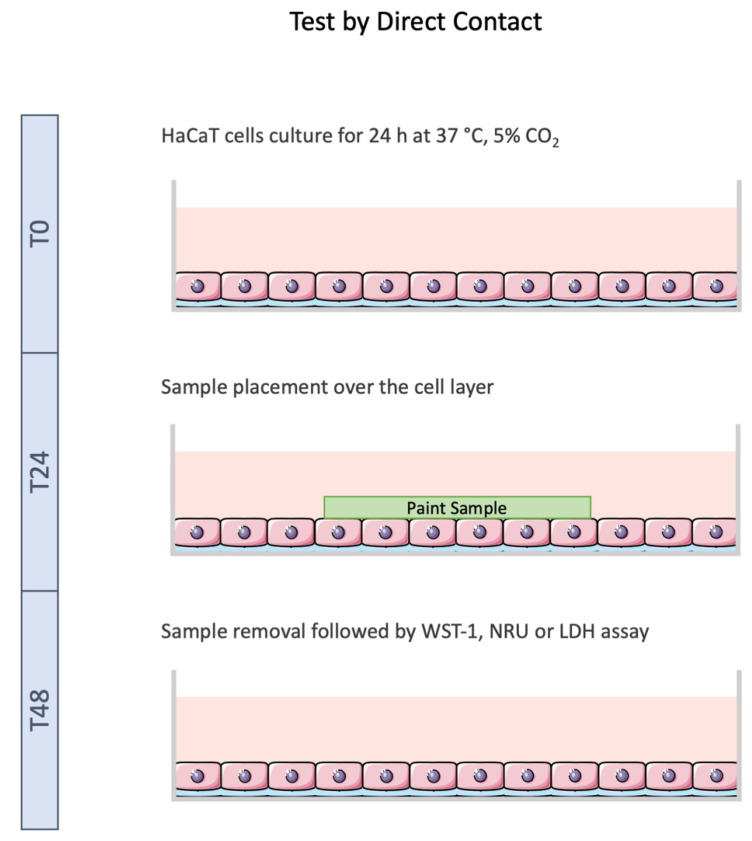
Schematic representation of test by direct contact performed with HaCaT cell line. Cells were exposed to the samples for 24 h followed by cytotoxicity assays. Image not to scale.

Tests by direct contact (Figure 1) were accomplished following ISO 10993-5 [9], with some modifications, and applied to the HaCaT cell line model.

Briefly, HaCaT cells were seeded in 6-well plates (1.0 × 10^5^ cells/mL) and allowed to adhere for 24 h at 37 °C, 5% CO_2_. Afterwards, complete medium was replaced with fresh assay medium (DMEM + 5% (*v*/*v*) FBS), and the samples (Un_Paint, TCS, ISB, W and Cu^2+^) were smoothly placed over the cell layer, in direct contact with cells. After, the cells were incubated for 24 h. Thereafter, the samples were smoothly extracted from the wells and cellular viability and membrane integrity were assessed as detailed in the following Section 2.6.1, Section 2.6.2 and Section 2.6.3.

During direct contact tests, microscopic observations were performed using an IT 400 inverted microscope by VWR (Radnor, PA, USA) in order to verify the cellular growth and morphology after 24 h of contact with the paint samples.

The exposures were carried out in three independent experiments, in triplicates.

### 2.5. Tests on Extracts

**Figure 2:**
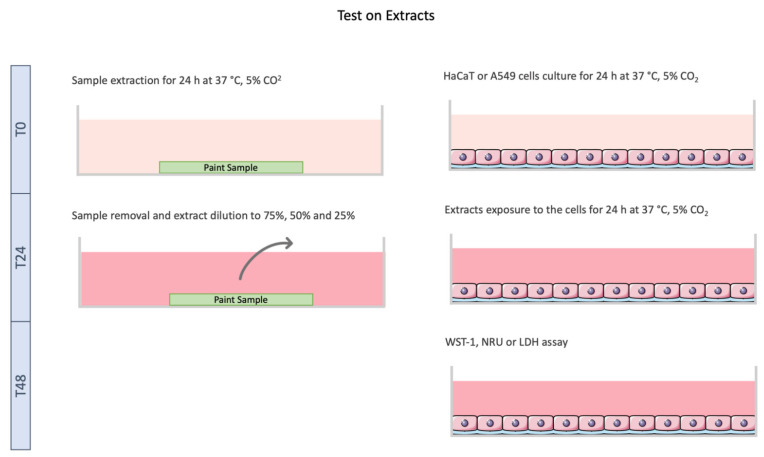
Schematic representation of test on extracts performed with HaCaT and A549 cell lines. Cells were exposed to the extracts for 24h followed by cytotoxicity assays. Image not to scale.

Tests on extracts (Figure 2) were performed according to ISO 10993-5 [9], with minor modifications. Briefly, the paint samples (10 × 10 mm) were deposited in 24 well-plates; 1 mL of assay medium (DMEM + 5% (*v*/*v*) FBS) was added to each well, followed by a 24 h incubation at 37 °C, 5% CO_2_, to allow the leaching of the chemicals from the samples.

Simultaneously, cells, both HaCaT and A549, were seeded in 96-well plates (1.0 × 10^5^ cells/mL) and incubated for 24 h at 37 °C, 5% CO_2_ in a humidified atmosphere to enable cell adhesion.

After 24 h of incubation, dilution series of the extracts were realized (100%, 75%, 50% and 25%). Then, the culture medium in the cells was replaced with the freshly prepared extracts’ dilutions, and cells were incubated for 24 h. Afterwards, cellular viability and membrane integrity were assessed.

The exposures were carried out in three independent experiments, in triplicates.

### 2.6. Citotoxicity Assays

#### 2.6.1. Cellular Viability (WST-1 Assay)

Cellular viability was determined by the colorimetric WST-1 Reagent Kit (Roche, Mannheim, Germany).

Briefly, after exposure for 24 h, the supernatant was removed and the cells (HaCaT or A549) were treated with 100 µL of WST-1 reagent (diluted 1:10 in serum-free medium) and incubated for 2h at 37 °C, 5% CO_2_, protected from light. Cells incubated only with assay medium (DMEM + 5% (*v*/*v*) FBS) were used as negative control, while cells treated with Triton X-100 solution (1% in assay medium) were used as positive control.

Optical density (OD) was measured at 450 nm (reference wavelength 630 nm) on a SpectraMax^®^ iD3 multi-mode microplate reader (Molecular Devices, San José, CA, USA). The blank sample OD consists of the OD measured in wells with all the vehicles used exposing the paint samples to the cells, but applied to empty wells, without cells. The blank negative control OD consists of the OD measured in wells with all the vehicles used exposing the negative control to the cells, but applied to empty wells, without cells. Higher OD values measured for the samples are associated with a higher cellular viability.

The results were expressed as percentage compared to the negative control, following Equation (1):

(1) $$\left(\frac{{\mathrm{OD}}_{\mathrm{sample}}-{\;\mathrm{OD}}_{\mathrm{blank}\;\mathrm{sample}}}{{\mathrm{OD}}_{\mathrm{negativecontrol}\;}-{\;\mathrm{OD}}_{\mathrm{blank}\;\mathrm{negative}\;\mathrm{control}}}\right)\times 100$$

#### 2.6.2. Cellular Viability (NRU Assay)

The NRU assay was performed according to ISO 10993-5 [9].

After exposure for 24 h, the supernatant was removed, and cells (HaCaT or A549) were treated with NRU solution (diluted 1:10 in serum-free medium) and incubated for 3h at 37 °C, 5% CO_2_, protected from light. Afterwards, the solution was discarded, and the cells were washed with PBS (100 µL/well). Then, the desorption solution composed of 49:50:1, water: absolute ethanol: acetic acid was added (200 µL/well), and the plate was shaken for 10 min, room temperature (RT). Cells incubated only with assay medium (DMEM + 5% (*v*/*v*) FBS) were used as negative control, while cells treated with SLS (0.2 mg/mL) were used as positive control. Optical density (OD) was measured at 540nm (reference wavelength 630 nm) on a SpectraMax^®^ iD3 multi-mode microplate reader (Molecular Devices, San José, CA, USA). Higher OD values measured for the samples are associated with higher cellular viability.

The results were expressed as percentage compared to the negative control, according to Equation (1).

#### 2.6.3. Membrane Integrity (LDH Assay)

Cellular membrane integrity was assessed using LDH (Lactate Dehydrogenase) assay through the Cytotoxicity Detection Kit (Roche, Mannheim, Germany).

Briefly, after exposure for 24 h, 100 µL of the supernatant was transferred to a 96-well plate and centrifuged at 250× *g* for 10 min at RT. After, 50 µL of supernatant was moved to a new 96-well plate and incubated with the LDH kit solution (in 1:1 ration) at RT, protected from light. Cells incubated only with assay medium (DMEM + 5% (*v*/*v*) FBS) were used as negative control, while cells treated with Triton X-100 solution (1% in assay medium) were used as positive control. After 20 min of incubation, the optical density (OD) was measured at 490 nm (reference wavelength 630 nm) on a SpectraMax^®^ iD3 multi-mode microplate reader (Molecular Devices, San José, CA, USA). The blank sample OD consists of the OD measured in wells with all the vehicles used exposing the paint samples to the cells, but applied to empty wells, without cells. The blank positive control OD consists of the OD measured in wells with all the vehicles used exposing the positive control to the cells, but applied to empty wells, without cells. Lower OD values measured for the samples are associated with lower LDH leakage from the cells.

The results were expressed as percentage compared to the positive control, following Equation (2):

(2) $$\left(\frac{{\mathrm{OD}}_{\mathrm{sample}}-{\;\mathrm{OD}}_{\mathrm{blank}\;\mathrm{sample}}}{{\mathrm{OD}}_{\mathrm{positivecontrol}\;}-{\;\mathrm{OD}}_{\mathrm{blank}\;\mathrm{positive}\;\mathrm{control}}}\right)\times 100$$

### 2.7. Genotoxicity Assays

#### 2.7.1. Cells Preparation for Alkaline Comet Assay

After growth of both cell lines (HaCaT and A549) for 24 h in 24-well plates (1.0 × 10^5^ cells/mL) the medium was replaced with a new complete medium containing two extracts’ concentrations (100% or 25%). Then, the plates were incubated for 24 h at 37 °C, 5% CO_2_. Cells incubated only with complete medium were used as negative control, and cells treated with a solution of MMS (800 µM) were used as a positive control. Three replicates of each condition were prepared.

After incubation, cells were washed with PBS and enzymatically dethatched using 0.25% trypsin-EDTA. Then, the cells were centrifuged for 5 min at 500× *g* and resuspended in a fresh culture medium with 10% DMSO. The cells were frozen and kept at −80 °C until comet assay was performed.

#### 2.7.2. Alkaline Comet Assay

The alkaline comet assay was conducted using a medium throughput 12-gel protocol, previously described with minor alterations [10]. The cells were thawed at 37 °C and counted in a Neubauer chamber using trypan blue dye to obtain a suspension of 10,000 cells in 1 mL of PBS that was then centrifuged for 5 min at 500× *g*. The pellet obtained was resuspended in 100 µL of 0.6% LMP agarose, and gels of 5 µL of each cell suspension (duplicates were made) were placed on microscope slides previously coated with 1% NMP agarose. The slides were placed for 5 min at 4 °C to allow the solidification of the gels and then were immersed for 1 h at 4 °C in cold lysis solution (NaCl 2.5 M, Na_2_EDTA 100 mM, Tris-base 10 mM, NaOH 10 M, pH 10, Triton-X 100 1%), protected from light.

After, the slides were washed for 5 min with cold PBS and immersed in electrophoresis solution (Na_2_EDTA 1 mM, NaOH 0.3 M, pH 13) for 30 min at 4 °C followed by electrophoresis that was performed for 30 min at 18V. Afterwards, for neutralization, the slides were washed with cold PBS and then with cold deionized water for 10 min each and fixed with ethanol 70% and ethanol 96% for 15 min each, being left to dry overnight.

Before microscopic evaluation, the slides were stained using SYBR^®^ Gold dye at 1:10,000 dilution of in TE buffer (Tris−HCl 10 mM and EDTA 1 mM, pH 7.5–8). The slides were observed using a Motic BA410 ELITE Series microscope, equipped with an EPI-fluorescence kit, with 100× magnification, and the comets were scored using the software Comet Assay IV image analysis software (Perceptive Instruments, Staffordshire, UK). At least 100 cells in each sample (50 cells/nucleoids in each gel) were scored.

#### 2.7.3. Cytokinesis-Block Micronucleus (CBMN) Assay

Micronucleus assay was performed as previously described by Rosário et al. with minimal alterations [11].

Previously sterilized glass slides (24 × 24 mm) were placed on 6-well plates. Cells were seeded at 2.5 × 10^5^/well concentration over the glass slides and incubated for 24 h at 37 °C, 5% CO_2_. Afterwards, the medium was replaced by fresh complete medium containing extracts (at 100% or 25%), and the plates were incubated for 24 h at 37 °C, 5% CO_2_.

Thereafter, cytochalasin B (2.5 ug/mL) was added to each well to block cytokinesis, and the plates were incubated for 29h. The medium was discarded, the cells were washed with PBS, and then cold (4 °C) absolute methanol was added for 15 min to fix the cells. The glass slides were collected and left to dry overnight.

Cells incubated only with complete medium were used as negative control, and cells treated with a solution of MMS (800 µM) were used as a positive control. Two replicates of each condition were prepared, and three independent experiments were performed.

For microscopic analysis, the slides were hydrated for 10 min in distilled H_2_O and then stained with acridine orange and mounted in slides. The slides were analyzed at Motic BA410 ELITE Series microscope equipped with an EPI-fluorescence kit.

The slides were scored for micronuclei (MNi), nucleoplasmic bridges (NPBs), nuclear buds (NBUDs) and nuclear division index (NDI) following the scoring criteria by Fenech [12]. MNi, NPBs and NBUDs were scored in 1000 binucleated cells per replicate. For NDI assessment, 1000 cells were scored (per replicate) for the number of nuclei present in the cell, with M1, M2, M3 and M4 being the number of cells with 1, 2, 3 or 4 nuclei.

NDI was then calculated using Equation (3), according to Eastmond and Tucker [13], with *N* being the total number of scored cells,

(3) $$\frac{M1+\left(M2\times 2\right)+\left(M3\times 3\right)+\left(M4\times 4\right)}{N}$$

### 2.8. Statistical Analysis

At least three replicates were used in each independent experiment that was repeated three times. The data from the three independent experiments was analyzed together. Data are reported as mean ± standard deviation (SD).

In the tests on extracts, tests by direct contact, alkaline comet assay and NDI, statistical significances of data against negative control were analyzed by one-way ANOVA followed by a Dunnett post hoc test.

In tests by direct contact statistical differences between different paints were analyzed by one-way ANOVA followed by Tukey’s test.

In alkaline comet assay and CBMN assay, the differences between different paints in the same extract’ concentration were analyzed by two-way ANOVA followed by Tukey’s test.

In CBMN assay, statistical significances of data against negative control were analyzed by two-way ANOVA followed by Dunnett multiple comparison test.

Statistical differences between the same concentration of the same paint, but for different cell lines (comparing A549 and HaCaT), were analyzed by two-way ANOVA followed by Sidak’s multiple comparison test.

Data were tested for normality and homogeneity of variances by Shapiro–Wilk and Bartlett’s tests, respectively.

The differences were considered statistically significant for *p* < 0.05. The statistical analyses were performed using Graph Pad Prism version 8.0 (GraphPad Software, San Diego, CA, USA, 2018).

## 3. Results

### 3.1. Tests by Direct Contact

**Figure 3:**
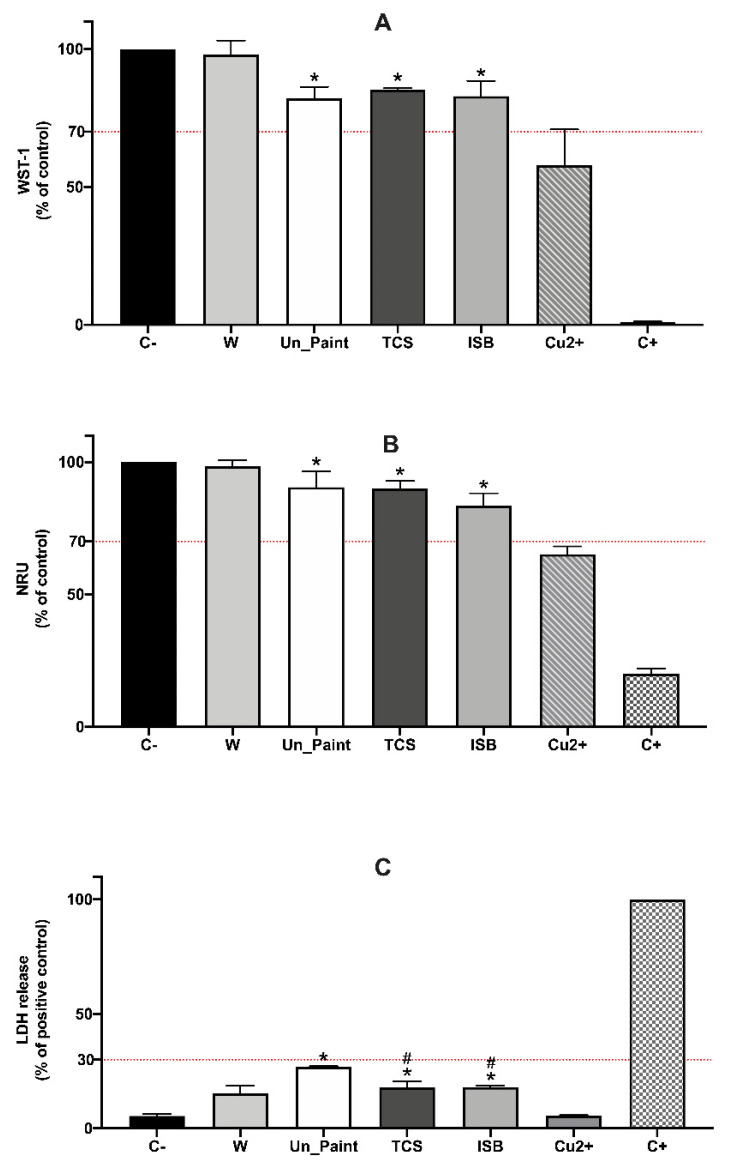
Results of WST-1 (**A**), NRU (**B**) and LDH (**C**) assays, with HaCaT cells, after 24 h of direct contact with the samples (in the x axis) of unmodified paint (Un_Paint), triclosan (TCS), isoborneol (ISB), transparent polymeric film (W) and Copper (Cu^2+^). C-negative control (assay medium), C+ positive control (Triton X-100 solution (1%) for WST-1 and LDH or SLS 0.2 mg/mL for NRU). The red lines represent the defined thresholds of acceptable values for each parameter, 70% for cellular viability and 30% for LDH leakage. The values are expressed as mean ± standard deviation. The statistical significance of samples compared to C- is represented by * and the statistical differences compared to Un_Paint are represented by ^#^ (One-way ANOVA; *p* < 0.05).

The tests by direct contact were realized using HaCaT cells to simulate a direct contact between the paints and the skin cells. Both WST-1 and NRU assays presented significant reductions on cell viability comparing to the negative control (Figure 3A,B). WST-1 results revealed a small but significant reduction on the cellular viability of TCS (14.79 ± 0.40%) and ISB (17.11 ± 3.97%). However, both TCS and ISB presented results with 85% and of 82% of cellular viability, respectively. TCS and ISB paints were not statistically different among themselves or from Un_Paint.

In NRU assay, the results were very similar to those obtained with WST-1. The two antimicrobial paints were statistically different from the negative control, however, with high values of cellular viability (over 83%). Once more, the TCS and ISB paints were nor statistically different among themselves or statistically different from Un_Paint.

Concerning the membrane integrity of the cells, LDH assay revealed a significant increase on LDH leakage after direct contact with the paints (Figure 3C). TCS and ISB presented lower values than Un_Paint (27 ± 0.31%), with 18% of LDH release for both paints. In this assay, TCS and ISB paints were not statistically different among themselves; however, both of them were statistically different from the Un_Paint paint.

During direct contact tests, microscopic observations were performed in order to verify the cellular growth and morphology after 24 h of contact with the paint samples (the images are in the Supplementary Materials, Figure S1). Comparing to the negative control, the presence of the paints did not alter the morphological aspect of the cells. However, a lower cellular density was verified when the paints were in contact with the cells, namely on areas more proximate to the paint samples.

### 3.2. Tests on Extracts

**Figure 4:**
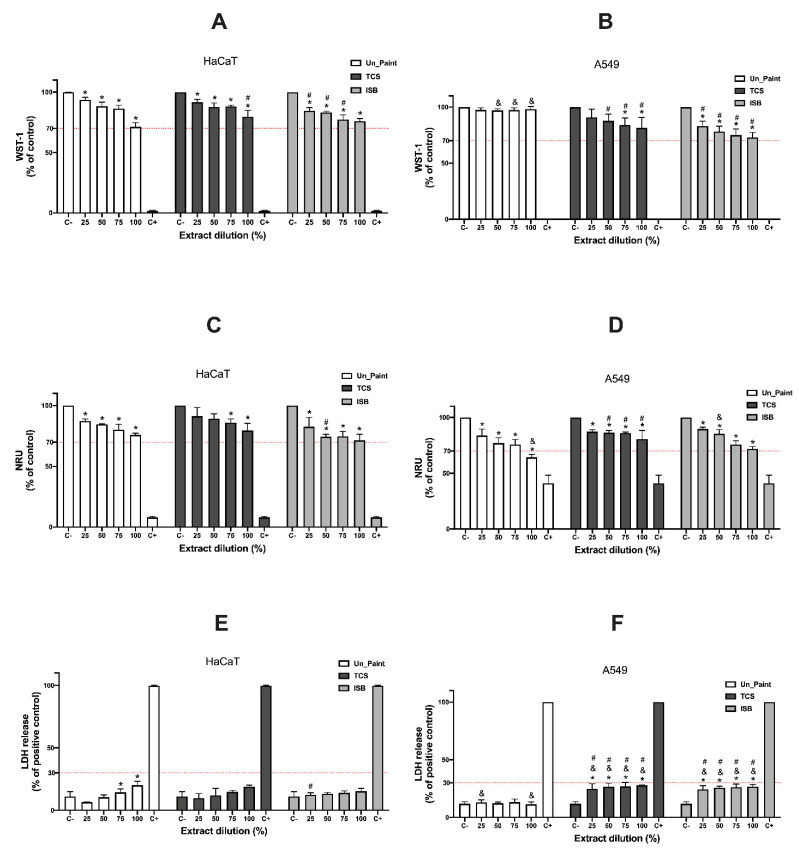
Results of WST-1 (**A**,**B**), NRU (**C**,**D**) and LDH (**E**,**F**) assays, with HaCaT and A549 cells, after 24 h incubation with the extracts (in the x axis) of the samples of Un_Paint, TCS and ISB extracts at concentration of 25, 50, 75 and 100%. C- negative control (assay medium), C+ positive control (Triton X-100 solution (1%) for WST-1 and LDH or SLS 0.2 mg/mL for NRU). The red lines represent the defined thresholds of acceptable values for each parameter, 70% for cellular viability and 30% for LDH leakage. The values are expressed as mean ± standard deviation. The statistical significance of the extracts compared to C- is represented by *, and the statistical differences compared to Un_Paint are represented by ^#^ (One-way ANOVA; *p* < 0.05). The statistical significance of the extract on A549 cells compared to the same extract, in the same concentration, on HaCaT cells is represented by ^&^ (Two-way ANOVA; *p* < 0.05).

Regarding the test on extracts performed with HaCaT cells (Figure 4A,C,E), the WST-1 assay revealed significant decreases on cell viability compared to the negative control. A decrease in HaCaT cells viability was observed when exposed to all extracts in all concentrations. TCS100% and ISB100% presented less pronounced reductions in cells viability (20.44 ± 4.25% and 24.08 ± 1.81%) compared to Un_Paint100% (28.79 ± 2.85%).

A similar trend was found with NRU assays; however, only IBS presented significant decreases for all tested concentrations. For TCS, only the higher concentrations (75% and 100%) were significantly different from the negative control.

In relation to the membrane integrity assay with HaCaT cells, the antimicrobial paints did not present statistical significance from the negative control.

Regarding the A549 cell line, the results of WST-1 assay presented significant decreases on cell viability for the higher concentrations of TCS (75% and 100%) and for all concentrations of ISB (Figure 4B). On the other hand, the results with A549 for NRU assay (Figure 4D) exhibited different results, since all the paints in all concentrations presented significant decreases in cell viability.

In the LDH assays with A549, TCS and ISB presented significant increases for all concentrations (Figure 4F).

### 3.3. Alkaline Comet Assay

Primary DNA damage in HaCaT and A549 cells was evaluated by alkaline comet assay. The chosen descriptor was % tail intensity, which measures the % of DNA in the tail [14].

According to the results obtained in cytotoxicity assays, we chose to perform the genotoxic assessment with both the minimum (25%) and maximum (100%) concentration of the extracts.

**Figure 5:**
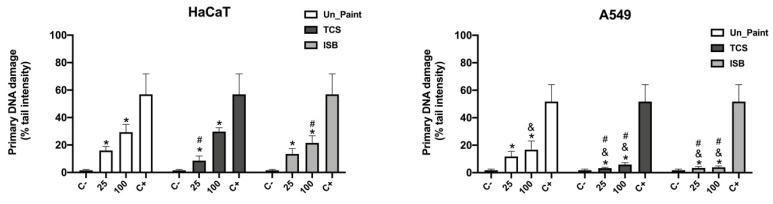
Results of alkaline comet assay, with HaCaT (left) and A549 (right) cell lines, after exposure to the extracts (in the x axis) of Un_Paint, TCS or ISB extracts for 24 h. C- negative control (complete medium), C+ positive control (MMS 800 µM solution). The values are expressed as mean ± standard deviation. The statistical significance of samples compared to C- is represented by * (One-way ANOVA; *p* < 0.05). The statistical significance of samples compared to Un_Paint is represented by # (Two-way ANOVA; *p* < 0.05). The statistical significance of the extract on A549 cells compared to the same extract, in the same concentration, on HaCaT cells is represented by ^&^ (Two-way ANOVA; *p* < 0.05).

Both antimicrobial paints presented significant increases in single-strand DNA breaks compared to the negative control (cells treated with complete medium), for both cell lines (Figure 5).

For HaCaT cells, TCS25% revealed a tail intensity of 8.63 ± 3.40% and TCS100% a tail intensity of 29.76 ± 2.82%. ISB25% presented a primary DNA damage of 13.44 ± 3.97% and ISB100% revealed a damage of 21.53 ± 5.16%. Both antimicrobial paints presented lower primary damages comparing to the Un_Paint (16.02 ± 2.90% for 25% extract and 29.35 ± 5.67% for 100% extract).

Regarding the A549 cells, TCS25% presented a primary damage of 3.28 ± 0.67% and TCS100% a damage of 5.93 ± 1.35%. ISB25 revealed a primary damage of 3.42 ± 0.93% and ISB100% a damage of 3.90 ± 1.00%. The antimicrobial paints presented values significantly lower than the Un_Paint (11.75 ± 3.72% for the 25% concentration and for 16.73 ± 6.30% for the 100% concentration).

### 3.4. Cytokinesis-Block Micronucleus (CBMN) Assay

**Figure 6:**
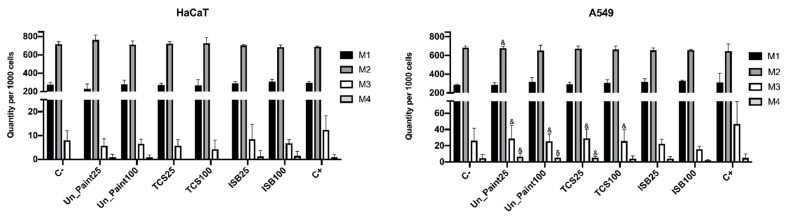
Quantity per 1000 cells of mononucleated (M1), binucleated (M2), trinucleated (M3) and tetranucleated (M4) cells of HaCaT (**left**) and A549 (**right**) cell lines, after exposure to extracts of the samples (in the x axis) Un_Paint, TCS or ISB extracts for 24 h. C- negative control (complete medium), C+ positive control (MMS 800 µM solution). The values are expressed as mean ± standard deviation. The statistical significance of samples compared to C- was analyzed by One-way ANOVA; *p* < 0.05. The statistical significance of samples compared to Un_Paint was analyzed by Two-way ANOVA; *p* < 0.05. The statistical significance of the extract on A549 cells compared to the same extract, in the same concentration, on HaCaT cells is represented by ^&^ (Two-way ANOVA; *p* < 0.05).

Cells incubated only with complete medium (negative control) presented a high number of binucleated cells (Figure 6), suggesting normal cell division, as expected. A residual number of MNi was detected for the negative control, and no NBUDs or NPBs were observed, both in HaCaT and A549 cell lines.

**Figure 7:**
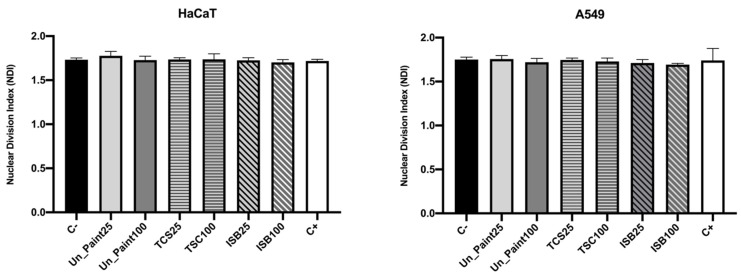
Nuclear division index (NDI) of HaCaT (**left**) and A549 (**right**) cell lines, after exposure to extracts of the samples (in the x axis) Un_Paint, TCS or ISB extracts for 24 h. C- negative control (complete medium), C+ positive control (MMS 800 µM solution). The values are expressed as mean ± standard deviation. The statistical significance of samples compared to C- was analyzed by One-way ANOVA; *p* < 0.05. The statistical significance of samples compared to Un_Paint was analyzed by Two-way ANOVA; *p* < 0.05. The statistical significance of the extract on A549 cells compared to the same extract, in the same concentration, on HaCaT cells was analyzed by Two-way ANOVA; *p* < 0.05.

After exposure with the paints’ extracts, the number of mononucleated and binucleated cells remained identical for both cell lines. This way, also the NDI calculated after exposure (Figure 7) presented similar values to the negative control (around 1.7).

Comparing the mitotic status of the two cell lines, statistical differences were only found on the number of trinucleated and tetranucleated cells on some extracts, TCS25% and TCS100%. The number of trinucleated and tetranucleated cells was higher in A549 cells.

**Figure 8:**
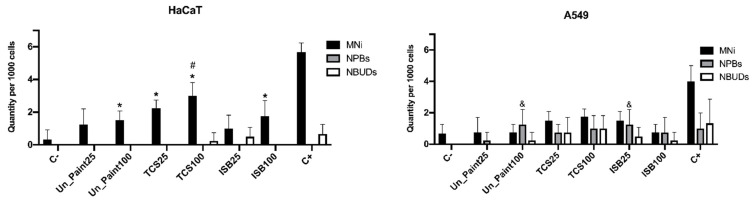
Micronuclei (MNi), nuclear buds (NBUDs) and nucleoplasmic bridges (NPBs) score on HaCaT (**left**) and A549 (**right**) cell lines, after exposure to extracts of the samples (in the x axis) Un_Paint, TCS or ISB extracts for 24 h. C- negative control (complete medium), C+ positive control (MMS 800 µM solution). The values are expressed as mean ± standard deviation. The statistical significance of samples compared to C- is represented by * (Two-way ANOVA; *p* < 0.05). The statistical significance of samples compared to Un_Paint is represented by # (Two-way ANOVA; *p* < 0.05). The statistical significance of the extract on A549 cells compared to the same extract, in the same concentration, on HaCaT cells is represented by ^&^ (Two-way ANOVA; *p* < 0.05).

For the HaCaT cell line, the occurrence of MNi in binucleated cells (Figure 8) increased with the exposure to paintsߣ extracts, with statistical significance for TCS25%, TCS100% and ISB100%. The antimicrobial paints only presented statistical difference from Un_Paint for the number of MNi in binucleated cells after exposure to TCS100%.

For A549 cells, a less pronounced increase in the occurrence of MNi was detected, however without present statistical differences from the negative control or from the Un_Paint. After exposure to the paints, NPBs and NBUDs were detected in A549 cells.

## 4. Discussion

Following the ISO 10993-5 criteria, a reduction over 30% in cell viability is considered cytotoxic, so we defined a limit of 70% of cell viability as acceptable for WST-1 and NRU assays and a limit of 30% cytotoxicity for LDH assay.

Despite using different biological endpoints, in general both WST-1 and NRU evaluate cellular viability. While WST-1 evaluates cells’ metabolic activity through mitochondrial enzymes, NRU uses lysosomes’ integrity as indicators of cell viability [15]. ISO 10993:5 suggest different cytotoxic colorimetric assays for quantitative evaluation of cellular viability, namely NRU and 3-(4,5-dimethylthiazol-2-yl)-2,5-diphenyl-2H-tetrazolium bromide (MTT). In our study we decided to use NRU assay, as suggested’ however, we decided to switch from MTT to WST-1 assay, since they are analogous tests and WST-1 involves less preparation steps, decreasing the occurrence of errors. Furthermore, MTT frequently presents some interferences, namely when testing materials [16,17]. In addition, we performed LDH assay to evaluate potential cytotoxicity towards cell membrane. This assay evaluates the leakage of LDH, a cytoplasmic enzyme, into the culture medium when the cells present damaged membranes [18].

In the test by direct contact, performed with HaCaT cells, results from WST-1 and NRU assays were very similar. In both assays, the paints presented an acceptable cytotoxic reaction with values of cellular viability above the established threshold of 70%, suggesting that the addition of the antimicrobial substances to the commercial paint does not increase its toxicity towards HaCaT cells, after direct contact. Regarding the microscopic observation of the cells after direct contact with the samples, the decrease in cellular density was verified in the presence of all paints. This occurrence suggests that this outcome may be related to the physical presence of the samples regardless of their constituents. This outcome was already verified in a similar study developed by Frewin et al. It is natural that the paint samples experience small undesired movements during preparation and incubation time with the cell layer, affecting cell adhesion on the closer areas of the samples or even removing some of the adhered cells on that area. As a result, cell density may be lower, especially closer to the samples, without affecting cellular viability [17].

In the tests on extracts, both viability assays performed with HaCaT cells revealed very positive results, with values of cellular viability above the established threshold of 70% for all paints in all concentrations. Besides, on both assays, HaCaT cells presented a dose-dependent response to the extracts’ exposure for the three paints. The obtained results, for LDH assay, were below the limit of 30%; TCS presented a maximum of 19% of LDH release and ISB a maximum of 15%. The paints with antimicrobial substances both presented low values of LDH leakage, suggesting a poor effect of those substances with HaCaT cells’ membrane.

For the tests on extracts performed on A549 cells, NRU assay showed a more prominent dose-dependent effect comparing to WST-1 assay. TCS and ISB displayed promising results, with values of cellular viability above 70% in both assays regardless of the concentrations. Additionally, the values of LDH release for TCS and ISB were below the 30% limit. In the LDH assay with A549 cells, no dose-dependent effect was observed, with LDH release values being very similar regardless of the extracts’ concentration. These results are in agreement with the ones found by Kwon et al., in a study using A549 cells to test the effect of chemicals mixtures, including TCS, where LDH release kept the same values independently of the concentration of the exposed chemical even if in MTT assay, a very marked dose-dependent effect was observed [19].

Regarding genotoxicity assessment, for HaCaT cells, the antimicrobial paints presented lower or similar values of primary DNA damage compared to the Un_Paint, used as reference. TCS presented a significantly different value of primary DNA damage for 25% concentration, compared to Un_Paint in the same concentration. For TCS100% the value was similar to the value of Un_Paint100%. ISB25% presented a lower value of tail intensity comparing to Un_Paint25%, without reachinh statistical significance. However, ISB100% presented a value of primary DNA damage significantly lower than Un_Paint100%.

Furthermore, a dose-dependent increase of DNA damage in HaCaT cells was observed for the antimicrobial paints, TCS and ISB, as assessed by the alkaline comet assay. A previous study by Wang et al. had already demonstrated that TCS induces DNA breaks in human cells (HepG2) and on a dose-dependent manner [20]. The values of DNA strand breaks found for ISB on HaCaT cells (13 and 22%) was also in accordance with a previous study involving other cell lines [21].

Concerning the A549 cells, both antimicrobial paints, TCS and ISB, in both concentrations, 25% and 100%, presented values of primary DNA damage significantly lower comparing to Un_Paint.

For A549 cells, the tested paints presented a less pronounced dose-dependent effect, and the primary DNA damage was lower compared to HaCaT cells. The comparison between cell lines presented statistical significance for TCS25%, TCS100%, ISB25% and ISB100%. For both antimicrobial paints, TCS and ISB, a very small increase (around 3% and 1% respectively) on DNA stand breaks was noticed between the 25% extracts concentration and the original extract.

A different sensitivity to the extract’s exposure was found between HaCaT and A549 cells. While in HaCaT cells a marked effect of extracts concentration was observed on the% tail intensity, with the DNA damage increasing with the increasing concentration of extracts for all paints, in A549 cells this effect was less evident.

A previous study from Horie and his colleagues [22] demonstrated that HaCaT and A549 cells have different behaviors towards the same exposure. In his work, comet assay was performed on both cell lines after similar exposure with a chemical. HaCaT cells presented an increase in DNA damage with increasing concentrations of the tested chemical, whereas A549 did not show a dose-dependent relation between the concentration of chemical and the DNA damage.

The obtained results suggest that the primary DNA damage is cell type-dependent, with different cell lines showing different susceptibility towards the same exposure. This way, different routes of exposure may represent distinct levels of risk.

According to the obtained results, ISB was the paint with lower DNA damage for the original extract (100%) in both cell lines. Slameňová et al. had already proved that borneol causes very little DNA damage on HepG2, (hepatocellular carcinoma), Caco-2 (colorectal adenocarcinoma) and VH10 (fore-skin fibroblasts) human cells with values of primary DNA damage of 10 to 20% depending on the chemical concentration and on the cell line [21].

Regarding the CBMN assay, the NDI after exposure to extracts presented similar values to the negative control for HaCaT cells. NDI is a biomarker for cell proliferation, which can be used to evaluate cytotoxicity [12]. Since no pronounced alterations were found on the mitotic status after exposure, with the proportion of mono and binucleated cells remaining similar, no big differences were expected on NDI. These results suggest that the exposure to the paints did not significantly affect the cellular division of HaCaT cells. After the exposure of the cells to paint extracts, the frequency of MNi increased. The occurrence of MNi is related to chromosome fragmentation and to whole chromosome loss. The MNi scoring is limited to binucleated cells to assure that the scored cells are dividing cells, able to express MNi [12]. This increase in MNi number was statistically significant for TCS25%, TCS100% and ISB100%. TCS100% also presented a number of MNi statistically different from Un_Paint100%. As expected, more concentrated extracts (100%) induced a higher number of MNi, evidencing a concentration gradient effect verified for the three substances (Un_Paint, TCS and ISB) when exposed to HaCaT cells. In this cell line, no NPBs were scored, and only a few NBUDs were detected. NBUDs are associated with amplified DNA elimination and with DNA repair complexes. NPBs are a biomarker of dicentric chromosomes that may result from DNA misrepair or from telomere end-fusions [12].

For A549 cells, the NDI after exposure to extracts also presented similar values to the negative control, as well as only slight alterations on the mitotic status. However, the number of MNi presented a tendency to increase after exposure to paints’ extracts even if without statistical significance. While on negative control no NPBs and no NBUDs were detected, after exposure to the paints, NPBs and NBUDs were scored, even if in very small amounts (without statistical differences from the negative control).

Comparing the two cell lines, A549 presented values statistically different from HaCaT only for the number of NPBs scored in ISB25%. However, in HaCaT cells, the increasing tendency in MNi occurrence after exposure was more pronounced than in the A549 cell line.

This study provides very relevant insights regarding the safety of antimicrobial paints to humans. Although the toxicity tests were performed in vitro, very useful outcomes were achieved as well as important information regarding the response of human cells towards the presence of the antimicrobial paints.

In vitro studies are the bottom line to assess toxicity, as these methods present several benefits in terms of ethical considerations and reduced costs. In an initial approach, in vitro testing, mimicking the in vivo and environmental conditions, is the most favorable option for a first screening of potential toxicity. In the future, more complex models, for example 3D cell models, may be used to obtain a more complex response towards the exposure to the paints.

Nonetheless, based on the obtained results, the incorporation of antimicrobial substances to the already commercialized paint did not increase its toxicity towards humans. This being said, we have sustained evidence that the antimicrobial paints may not represent an additional risk of human exposure to the paints.

## 5. Conclusions

After direct contact for 24 h with the developed paints containing the antimicrobial substances, TCS and ISB, the skin cells HaCaT presented acceptable values of cellular viability (>70%) and of LDH leakage (<30%). Likewise, both cell lines, HaCaT and A549, presented non-toxic results of cellular viability and membrane integrity after exposure to the extracts produced from the self-disinfecting paints.

Regarding the genotoxicity assessment, the antimicrobial paints revealed primary DNA damages equal to or lower than the reference paint (Un_Paint). The cellular division was not affected by the presence of the antimicrobial substances on the paint, and the number of MNi, NPBs and NBUDs was very low, even if statistically significant from the negative control.

Summing up, our study demonstrates that self-disinfecting paints, containing TCS or ISB in low concentrations, present levels of cytotoxicity within the acceptable limits suggested by ISO 10993 for both cell lines models HaCaT and A549. Moreover, our study shows that the genotoxicity of these paints is not significantly affected due to the addition of the substances TCS and ISB.

This way, it is possible to conclude that self-disinfecting paints with TCS or ISB may be an efficient and safe strategy to prevent surface colonization with microorganisms in locals prone to infection spreading. Moreover, this antimicrobial coating, with the necessary adaptations, may have an important range of applications, from medical devices to microbiologically susceptible environments.

## Data Availability

The data presented in this study are available on request from the corresponding author.

## Supplementary Materials

The following are available online at https://www.mdpi.com/article/10.3390/toxics10020058/s1, Figure S1: Microscopic images (100× magnification) of the HacaT cells with complete medium used as negative control (C-) or after 24 h of incubation in direct contact with the samples Unmodified paint (Un_Paint), Triclosan (TCS), Isoborneol (ISB), Transparent polymeric film (W) and Cooper (Cu^2+^).
