# Supplementary material for: In Vitro Cyto- and Genotoxicity Assessment of Antibacterial Paints with Triclosan and Isoborneol
Source: Toxics. 2022 Jan 27;10(2):58. doi: 10.3390/toxics10020058 (PMC8877867; doi:10.3390/toxics10020058)
Supplement: Supplementary file 1 [file toxics-10-00058-s001.zip › toxics-1550938-supplementary.pdf]

toxics

Supplementary Materials: In Vitro Cyto- and Genotoxicity Assessment of Antibacterial Paints with Triclosan and Isoborneol

Micaela Machado Querido, Fernanda Rosário, Maria João Bessa, Francisca Mendes, José Carlos Teixeira, João Paulo Teixeira and Cristiana Costa Pereira

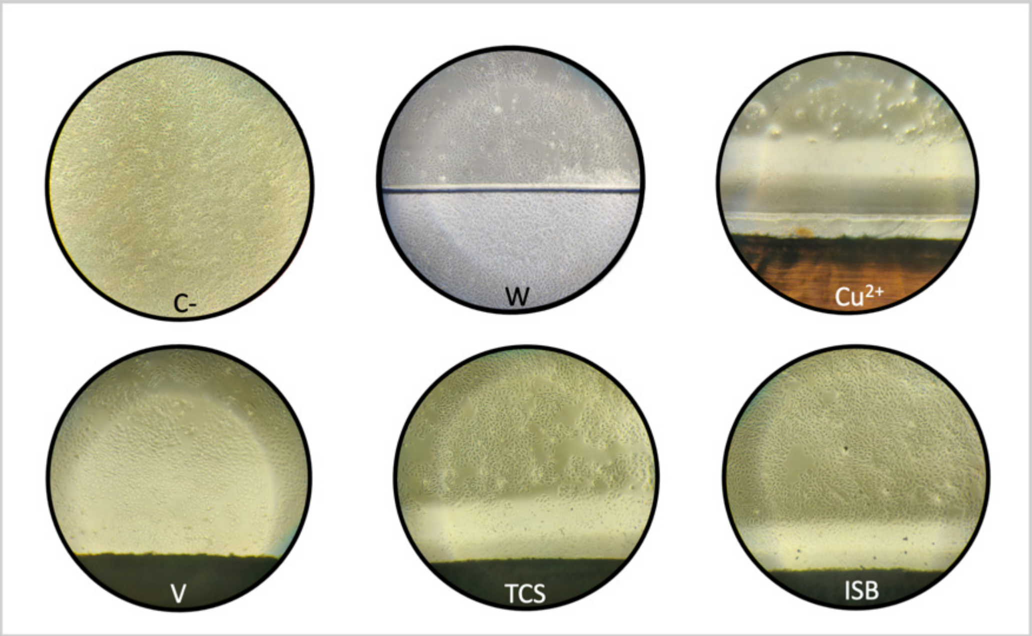

Figure S1. Microscopic images (100× magnification) of the HacaT cells with complete medium used as negative control (C-) or after 24h of incubation in direct contact with the samples Unmodified paint (Un_Paint), Triclosan (TCS), Isoborneol (ISB), Transparent polymeric film (W) and Cooper (Cu^{2+}).
